# 72 hour Holter monitoring, 7 day Holter monitoring, and 30 day intermittent patient-activated heart rhythm recording in detecting arrhythmias in cryptogenic stroke patients free from arrhythmia in a screening 24 h Holter

**DOI:** 10.1515/med-2020-0203

**Published:** 2020-07-18

**Authors:** Andrzej Kułach, Milena Dewerenda, Michał Majewski, Anetta Lasek-Bal, Zbigniew Gąsior

**Affiliations:** Department of Cardiology, Medical University of Silesia, Ziolowa 47, 40-635 Katowice, Poland; Department of Neurology, Upper-Silesian Medical Center, Katowice, Poland; Department of Cardiology, Upper-Silesian Medical Center, Katowice, Poland; Department of Neurology, Medical University of Silesia, Katowice, Poland

**Keywords:** atrial fibrillation, cryptogenic ischemic stroke, Holter ECG, patient-activated ECG recorder, supraventricular arrhythmia, SVT runs

## Abstract

**Introduction:**

According to recent studies, silent atrial fibrillation (AF) is a common cause of cryptogenic ischemic stroke (CIS). 12-lead electrocardiogram (ECG) and 24 h Holter are not efficient to reveal an occult arrhythmic cause of stroke.

**Objectives:**

The aim of the study was to evaluate 72 h Holter, 7 day Holter monitoring, and intermittent single-lead ECG recording in patients with CIS to identify cases with the arrhythmic cause of stroke in patients with CIS in whom 24 h ECG Holter was free from arrhythmia.

**Methods:**

72 patients (aged 60 ± 9 years, 44 males) with CIS and no arrhythmic findings in 24 h Holter were enrolled. All patients had 7 day Holter monitoring and received handheld ECG recorder (CheckMe, Viatom) for ambulatory 30 ± 3 days ECG recording. AF, supraventricular tachycardia (SVT runs of ≥5 QRS), and other arrhythmias were assessed in the first 72 h of Holter recording, in 7 day-recording, and in handheld ECG strips.

**Results:**

72 h-recording revealed AF in four cases (5.6%) and SVT in 18 (25%) cases. 7 day Holter confirmed AF in seven patients (10%) and SVT in 27 patients (37.5%). There was no difference in regards to CHADS2VASc score between patients with SVT and non-arrhythmic group (3.6 ± 1.1 vs 3.4 ± 1.6; *p* = NS). Symptoms did not correlate with findings. Patient-activated handheld ECG recorders were used with good compliance. The mean number of recordings was 49 ± 30. Except for PACs, there was only one case of AF documented in 3,531 strips.

**Conclusions:**

7 day Holter performs better than 72 h and reveals supraventricular arrhythmias in every third and AF in 10% of CIS patients who were free from arrhythmia in 24 h ECG monitoring. 30 day intermittent ECG monitor does not yield diagnostic value in CIS.

## 1 Introduction

An origin of an acute stroke remains unknown in 20–40% [1]. Recent studies show that in many cases of cryptogenic ischemic stroke (CIS), the cause is attributable to silent atrial fibrillation (silent AF) [2,3]. The prevalence of AF in cryptogenic stroke population ranges from below 10% to >25% depending on the timing, duration, and method of monitoring [4].

Paroxysmal AF is often asymptomatic and is likely to be undetected in patients with ischemic stroke [5]. Other supraventricular arrhythmias, including multiple PACs and particularly short supraventricular tachycardia (SVT) runs, seem to be an important predictor of AF and recurrent stroke [21].

Considering AF is a leading preventable cause of recurrent stroke, detection of AF after cryptogenic stroke is crucial for further therapy and prognosis. Despite current guidelines recommending >48–72 h recording for AF screening after stroke, a 12-lead electrocardiogram (ECG) and 24 h Holter monitoring is a routine screening tool in stroke survivors.

It is well recognized that the longer the time of monitoring is, the higher the prevalence of detected arrhythmic episodes [6]. The most precise data on AF prevalence in stroke survivors come from studies in pacemaker/implantable cardioverter-defibrillator (ICD) patients [7] and from studies utilizing implantable loop recorders (ILRs) [8]. While cardiac implantable electric devices (CIEDs) are a source of valuable data, they are not a diagnostic option in the general population for obvious reasons. On the contrary, ILRs are good diagnostic tools, but the cost and invasive procedure of implantation make the tool less acceptable for both patients and healthcare systems.

Less sensitive but more available and affordable options include extended (>48–72 h) Holter monitoring, continuous ECG recording with telemetry, loop recorders and patient activated (noncontinuous) repeated ambulatory ECG recorders.

7 day non-invasive ECG continuous monitoring is commonly available and despite some drawbacks – acceptable for patients. ECG recorders of longer duration are rarely available.

Patient-activated, handheld, single-lead ECG event recorders have been proposed as a useful tool in symptomatic patients (dizziness, palpitations), and some clinical settings are even more cost-effective than conventional 24 h or 48 h Holter-ECG monitoring [9,10,11]. Due to low cost and good specificity in arrhythmia detection, they are being tested in many conditions where a proper diagnosis is crucial for further treatment. It seems that more than one modality should be used to increase the rate of confirmed AF in poststroke patients. Any effort should be made to identify the groups, which would benefit from a broader and more detailed diagnostic approach to seek occult arrhythmia.

The aim of the study was to evaluate 72 h Holter, 7 day Holter monitoring, and intermittent single-lead ECG monitoring strategy to identify cases with AF and SVT runs in patients with CIS in whom 24 h Holter was negative for arrhythmia.

Additionally, correlation of symptoms, clinical characteristics, and echocardiographic findings were evaluated.

## 2 Patients and methods

We analyzed 72 patients (aged 59 ± 7 years, 44 males) with ischemic stroke with no significant disability and cognitive impairment. In all patients, carotid artery stenosis and any relevant arrhythmic findings in 24 h Holter (i.e., supraventricular runs ≥5 QRS, AF) were excluded.

The patients were also screened for other common non-arrhythmic causes of cardiac thromboembolism.

Patients were enrolled in the study 4–7 days after admission to the stroke unit. In all patients, we performed the echocardiographic study, and parameters of the left atrium (LA diameter, area, and volume) were recorded. Patients had 7 day Holter (Lifecard CF/Pathfinder SL, Reynolds Medical, USA) monitoring and received handheld ECG recorder (CheckMe Pro, Viatom, PRC) for ambulatory 30 ± 3 days single-lead ECG recording (30 s measurements twice a day plus whenever symptomatic).

We recorded AF episodes (duration >30 s), SVT (runs of ≥5 QRS), and other arrhythmias in the first 72 h and in the 7 day recording. ECG strips from portable recorders were assessed manually by two cardiologists to identify any supraventricular and ventricular arrhythmias.

### 2.1 Statistical analysis

Statistical analysis was performed with Statistica 13.1 (Dell). All values were expressed as average (SD). Differences were considered to be significant at *p* < 0.05. In order to check the normality of the distribution, the Shapiro–Wilk test was performed. In case of a normal distribution, the Student *t*-test was performed; otherwise, the Mann–Whitney *U* test was used. Qualitative parameters were compared using Pearson’s chi-square and McNemar’s test. The study was accepted by the Ethics Committee of the Medical University of Silesia. The investigation is in line with the principles outlined in the Declaration of Helsinki. All patients gave written informed consent for participation in the study.

## 3 Results

The baseline characteristics of the studied group are presented in Table 1.

**Table 1:** Baseline characteristics of the studied group

Parameter	
Age (years), mean ± SD	59.7 ± 9.1
CHADS2-VASC score, mean ± SD	3.53 ± 1.6
Congestive heart failure, *n* (*n*%)	2 (3%)
Ejection fraction (%), mean ± SD	59 ± 4
Mitral regurgitation mild/moderate/severe	29 (40%)/6 (8%)/0 (0%)
Hypertension, *n* (*n*%)	37 (51%)
Diabetes, *n* (*n*%)	10 (14%)
Vascular disease	5 (7%)

Numerical data are presented as mean ± SD (standard deviation) or *n* (*n*%).

7 day Holter revealed SVT (runs ≥5 QRS) in 27 patients (37.5%). SVT duration was 5–164 beats; rate 120–188 bpm. In 7 of 27 SVT patients, AF episodes (duration 40 s – 7 min) were found. Premature atrial complexes (PACs) were common in the studied group; yet, only 14 patients (19%) had >100 PACs/day. Besides, 27 patients (37.5%) presented bradycardia (<50 beats per minute), and one of them required pacemaker implantation for the brady-tachy syndrome.

**Figure 1:**
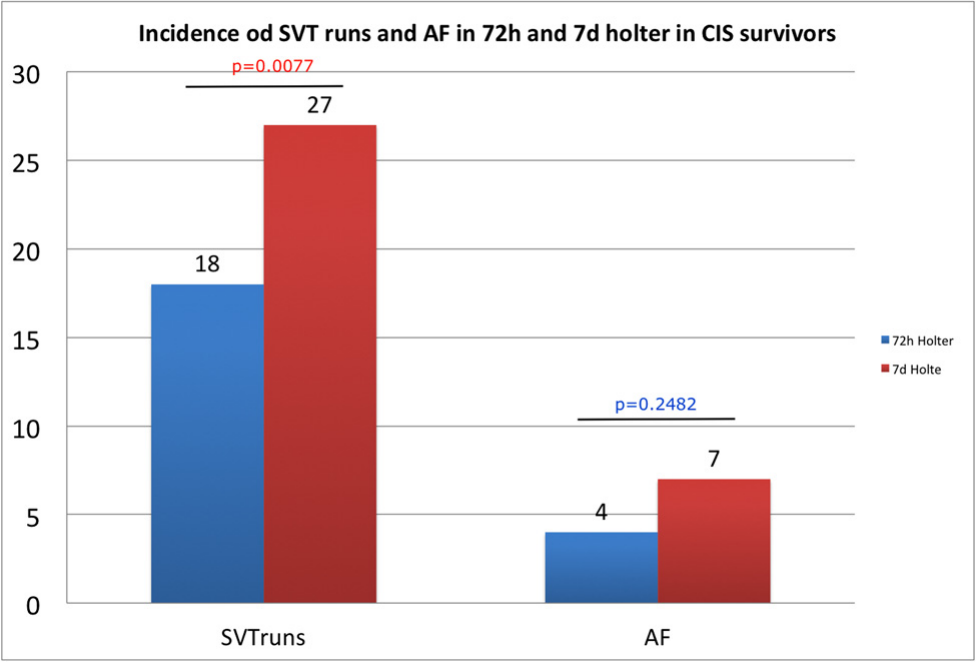
Incidence of SVT runs and AF in 72 h and 7 days Holter in cerebral ischemic stroke survivors.

In the analysis of initial 72 h, AF was recorded in four subjects (4(5.6%) vs 7(9.7%); *p* = 0.2482), while SVT in 18 patients (18 (25%) vs 27 (37.5%); *p* = 0.0077), which is less than in 7 day recording (Figure 1).

Based on 7 day Holter results, patients were classified into two groups: patients without SV arrhythmia and patients with SV arrhythmia (SV runs of ≥5QRS or AF). Selected parameters are shown in Table 2.

**Table 2:** Selected echocardiographic and clinical parameters

	No SV arrhythmia (*n* = 45)	SV arrythmia (*n* = 27)	AF (*n* = 7; subgroup of SV arrhythmia)
Age (years)	59 ± 9	61 ± 10	66 ± 8
CHADS2-VASC score	3.4 ± 1.6	3.6 ± 1.1	3.9 ± 1.1
Palpitations (*n*, *n*%)	15 (33%)	7 (26%)	2 (29%)
Beta-blocker (*n*, *n*%)	11 (24%)	12 (44%)	3 (43%)
Left atrium diameter (mm)	38 ± 6	37 ± 9	39 ± 7
Left atrium volume (ml)	62 ± 26	58 ± 17	64 ± 17

Numerical data are presented as mean ± SD. No significant differences between groups were observed. SV, supraventricular; AF, atrial fibrillation.

Patients did not differ with regard to age and echocardiographic parameters (ejection fraction, significant valvular disease, and particularly left atrium parameters). CHADS2VASc score was similar in SV arrhythmia and non-arrhythmic group (3.6 ± 1.1 vs 3.4 ± 1.6; *P* = ns).

Twenty two patients (31%) complained of palpitations during the study or in history. However, the symptom did not correlate with tachycardia events – only seven symptomatic subjects had SVT or AF in 7 day Holter. Two out of seven patients with confirmed AF and only 7 out of 27 SVT patients declared symptoms. Beta-blockers use was higher in the arrhythmic group; yet, only two of the patients with confirmed AF was treated with a beta-blocker.

Patient-activated handheld ECG recorders were properly used with no apparent operational issues. 97% of ECG strips was of good quality. The mean number of ECG strips was 49 ± 30; seven patients (10%) recorded less than 30 strips, and 13 (18%) – less than 50. Patients who declared palpitations recorded more strips (78 ± 30 vs 44 ± 17; *p* < 0.05), but except for rare PACs, and one case of symptomatic AF, also recorded in 7 day Holter, there were no documented arrhythmic events in 3,531 strips. All records were reviewed manually by two cardiologists.

## 4 Discussion

Prevalence of AF in cryptogenic stroke survivors is strictly related to modality and duration of ECG recording and observation. In contrast to the previous statement, recent European Society of Cardiology guidelines for the management of AF (2016) recommends repeated ECG recordings, extended Holter monitoring (>48–72 h) and both non-invasive and implantable recorders in stroke survivors to identify cases with an underlying arrhythmic cause [12]. In a real-world setting, 24 h Holter is still a basic screening for AF after stroke.

ILRs are a golden standard in searching for AF. In CRYSTAL-AF study (Reveal XT ICM, Medtronic, in cryptogenic stroke patients), rates of AF detection were 8.9% after 6 months, 12.4% after 12 months, and 30% after 36 months. Importantly, the median time to AF detection was 41 days during the first 6 months follow-up and went up to 84 days when follow up was extended to 3 years [8]. This may put in doubt strategies that focus on shorter monitoring duration. However, in the EMBRACE study, AF detection rate for 30 days external loop recorder was 16.1% in post-stroke patients [6].

Data from CIED patients suggests that the incidence of atrial high-rate episode (AHRE) in stroke survivors reaches 30–45% in 30 months follow-up. However, data from ASSERT and TRENDS studies suggest that it is AF episodes duration and AF burden that matters with regard to the risk of stroke [7,13]. Short and infrequent episodes are unlikely to increase thromboembolic risk, especially in patients with low clinical risk factor profile. Additionally, it is worth noting that data of CIED patients cannot be directly translated to the general population. Both pacemaker patients (mostly implanted due to sinus node dysfunction) and ICD patients (due to heart failure) are more likely to have AF than the general population.

In a real-world setting, there is no reasonable tool for non-invasive and long-term monitoring for AF.

The cost of implantable monitoring devices and invasive procedure of implantation rise the question, whether commonly used, less expensive, and noninvasive forms of monitoring can be a reliable alternative for continuous monitoring.

24 h Holter yields a detection rate of 1–2% in stroke survivors group. Jabaudon et al disclosed AF in 5% using 24 h Holter, then another 5.7% using 7 day recording [14]. Wachter and co-workers identified AF in 16% of CIS survivors using a strategy of repeated 10 day Holter (three rounds within 6 months follow up) vs standard care [15]. Recently, in order to make long-term monitoring more acceptable for patients, wearable or patch-like recorders are being developed. Data from wearable ECG patch (ZioPatch, iRhythm) seem reliable, but in a cohort of patients screened for AF due to stroke in recent history, a detection rate for AF was 5%, with a median recording duration of 13 days [16].

We enrolled patients, in whom 24 h Holter did not show any significant arrhythmia. 72 h analysis performed significantly better (four cases of AF, 18 cases with SVT), and 7 day recording doubled the number of identified arrhythmic cases. Considering the short observation period, AF in 10% cases seems high, but this might be a matter of the population (mean age 60), and the definition of AF (AF > 30 s, but only three of them >2 min). Apart from searching for AF, we have focused on other supra-ventricular arrhythmias, particularly SV runs) that are thought to predispose to AF or coincide with AF [18,19]. In the analysis of patients from prospective FindAF trial performed by Weber-Krüger et al. [21], patients with stroke who had SVT runs (at least 5 QRS, but <30 s) were more likely to have a recurrent stroke and showed 2.5 times more novel AF in 3 year follow than the controls. This suggests that the group will need further follow-up and repeated monitoring. As our study shows, SVT runs are observed in a large proportion of patients with CIS and may be found in every fourth patient in 72 h monitoring and reach 37% in 7 day Holter in patients in whom 24 h Holter would have missed this finding.

Although there are many discrepancies, it is equivocal that any type of extended ECG monitoring is significantly better than a standard 24 h Holter. More studies including long-term telemetric monitoring are ongoing [17].

In our study, patients with supraventricular arrhythmias did not differ from the nonarrhythmic group with regard to age, CHADS2VASc score, or echo parameters of the left atrium. Also, we did not find any correlation between arrhythmia and declared symptoms. Considering the silent nature of AF in stroke, it is not surprising and consistent with available literature. In CRYSTAL-AF, more than 3/4 patients with detected AF were asymptomatic.

Screening for AF with patient activated leadless ECG monitor has been successfully used in symptomatic patients (palpitations) and in general population with particular risk factors [9]. The most common protocol is two recordings per day for 1 month and additional records when symptomatic. We used the same protocol. In our study, we have found only one case of arrhythmia in more than 3,500 strips. In recently published REHEARSE AF study investigators claim four times higher detection of AF in intermittent ECG recording group (AliveCor Kardia, two records/day, duration 1 year, general population), but absolute numbers of diagnosed cases were 19/500 vs 5/501 patients, which means it requires 71 patients to perform two measurements/day for 12 months to find one case of AF [20]. In our opinion in asymptomatic patients without known arrhythmia intermittent, patient-activated ECG monitors do not yield diagnostic values.

In summary, our diagnostic approach with 7 day Holter and 1 month intermittent ECG monitoring; let us identify AF in 10% of CIS survivors with 24 h Holter free from arrhythmia, and indicated that 37% of these patients might require further investigation due to arrhythmia predisposing to AF. We would like to stress that the strategy is low cost and accessible. Handheld patient-activated ECG recorder fails to prove usefulness in these settings.

## 5 Conclusions

In stroke survivors with no arrhythmic findings in 24 h Holter, extended monitoring may reveal AF in up to 10% and SVT runs in over one-third of the population. 7 day Holter performs better than the guideline-recommended 72 h recording.

The arrhythmic findings do not correlate with symptoms and patients with arrhythmia do not differ with regard to CHADS2VASc score and to left atrium parameters from patients with no arrythmia.

Despite reasonable compliance and quality of ECG obtained, patient-activated handheld ECG recording (30 day) does not yield diagnostic values in patients with CIS.

## References

[1] Li L, Yiin GS, Geraghty OC, Schulz UG, Kuker W, Mehta Z, et al. Oxford Vascular Study. Incidence, outcome, risk factors, and long-term prognosis of cryptogenic transient ischaemic attack and ischaemic stroke: a population-based study. Lancet Neurol. 2015;14:903–13. 10.1016/S1474-4422(15)00132-5 PMC5714616 26227434

[2] Adams HP Jr, Bendixen BH, Kappelle LJ, Biller J, Love BB, Gordon DL, et al. Classification of subtype of acute ischemic stroke. Definitions for use in a multicenter clinical trial. TOAST Stroke. 1993;24:35–41. 10.1161/01.str.24.1.35 7678184

[3] Hart RG, Diener HC, Coutts SB, Easton JD, Granger CB, O'Donnell MJ, et al. Embolic strokes of undetermined source: the case for a new clinical construct. Lancet Neurol. 2014;13:429–38. 10.1016/S1474-4422(13)70310-7 24646875

[4] Kishore A, Vail A, Majid A, Dawson J, Lees KR, Tyrrell PJ, et al. Detection of atrial fibrillation after ischemic stroke or transient ischemic attack: a systematic review and meta-analysis. Stroke. 2014;45:520–6. 10.1161/STROKEAHA.113.003433 24385275

[5] Ziegler PD, Glotzer TV, Daoud EG, Singer DE, Ezekowitz MD, Hoyt RH, et al. Detection of previously undiagnosed atrial fibrillation in patients with stroke risk factors and usefulness of continuous monitoring in primary stroke prevention. Am J Cardiol. 2012;110:1309–14. 10.1016/j.amjcard.2012.06.034 22819433

[6] Gladstone DJ, Spring M, Dorian P, Panzov V, Thorpe KE, Hall J, et al. Atrial fibrillation in patients with cryptogenic stroke. N Engl J Med. 2014;370:2467–77. 10.1056/NEJMoa1311376 24963566

[7] Daoud EG, Glotzer TV, Wyse DG, Ezekowitz MD, Hilker C, Koehler J, et al. Temporal relationship of atrial tachyarrhythmias, cerebrovascular events, and systemic emboli based on stored device data: a subgroup analysis of TRENDS. Heart Rhythm. 2011;8:1416–23. 10.1016/j.hrthm.2011.04.022 21699833

[8] Sanna T, Diener HC, Passman RS. CRYSTAL AF Investigators. Cryptogenic stroke and underlying atrial fibrillation. N Engl J Med. 2014;370:2478–86. 10.1056/NEJMoa1313600 24963567

[9] de Asmundis C, Conte G, Sieira J, Chierchia GB, Rodriguez-Manero M, Di Giovanni G, et al. Comparison of the patient-activated event recording system vs traditional 24 h Holter electrocardiography in individuals with paroxysmal palpitations or dizziness. Europace. 2014;16:1231–5. 10.1093/europace/eut411 24574492

[10] Kaleschke G, Hoffmann B, Drewitz I, Steinbeck G, Naebauer M, Goette A, et al. Prospective, multicentre validation of a simple, patient-operated electrocardiographic system for the detection of arrhythmias and electrocardiographic changes. Europace. 2009;11:1362–8. 10.1093/europace/eup262 19797150

[11] Tieleman RG, Plantinga Y, Rinkes D, Bartels GL, Posma JL, Cator R, et al. Validation and clinical use of a novel diagnostic device for screening of atrial fibrillation. Europace. 2014;16:1291–5. 10.1093/europace/euu057 PMC4149608 24825766

[12] Kirchhof P, Benussi S, Kotecha D, Ahlsson A, Atar D, Casadei B, et al. ESC Scientific Document Group. 2016 ESC guidelines for the management of atrial fibrillation developed in collaboration with EACTS. Eur Heart J. 2016;(37):2893–962. 10.1093/eurheartj/ehw210 27567408

[13] Healey JS, Connolly SJ, Gold MR, Israel CW, Van Gelder IC, Capucci A, et al. Subclinical atrial fibrillation and the risk of stroke. N Engl J Med. 2012;366:120–9. 10.1056/NEJMoa1105575 22236222

[14] Jabaudon D, Sztajzel J, Sievert K, Landis T, Sztajzel R. Usefulness of ambulatory 7 day ECG monitoring for the detection of atrial fibrillation and flutter after acute stroke and transient ischemic attack. Stroke. 2004;35:1647–51. 10.1161/01.STR.0000131269.69502.d9 15155965

[15] Wachter R, Gröschel K, Gelbrich G, Hamann GF, Kermer P, Liman J, et al. Holter-electrocardiogram-monitoring in patients with acute ischaemic stroke (Find-AF(RANDOMISED)): an open-label randomised controlled trial. Lancet Neurol. 2017;16:282–90. 10.1016/S1474-4422(17)30002-9 28187920

[16] Tung CE, Su D, Turakhia MP, Lansberg MG. Diagnostic yield of extended cardiac patch monitoring in patients with stroke or TIA. Front Neurol. 2014;5:266. 10.3389/fneur.2014.00266 PMC4290477 25628595

[17] Kalarus Z, Balsam P, Bandosz P, Grodzicki T, Kaźmierczak J, Kiedrowicz R, et al. NOninvasive monitoring for early detection of atrial fibrillation: rationale and design of the NOMED-AF study. Kardiol Pol. 2018;76:1482–5. 10.5603/KP.a2018.0193 30211437

[18] Gladstone DJ, Dorian P, Spring M, Panzov V, Mamdani M, Healey JS, et al. Atrial premature beats predict atrial fibrillation in cryptogenic stroke: results from the EMBRACE trial. Stroke. 2015;46:936–41. 10.1161/STROKEAHA.115.008714 25700289

[19] Kamel H, Elkind MS, Bhave PD, Navi BB, Okin PM, Iadecola C, et al. Paroxysmal supraventricular tachycardia and the risk of ischemic stroke. Stroke. 2013;44:1550–4. 10.1161/STROKEAHA.113.001118 PMC3950597 23632982

[20] Halcox JPJ, Wareham K, Cardew A, Gilmore M, Barry JP, Phillips C, et al. Assessment of remote heart rhythm sampling using the aliveCor heart monitor to screen for atrial fibrillation: the REHEARSE-AF study. Circulation. 2017;136:1784–94. 10.1161/CIRCULATIONAHA.117.030583 28851729

[21] Weber-Krüger M, Lutz C, Zapf A, Stahrenberg R, Seegers J, Witzenhausen J, et al. Relevance of supraventricular runs detected after cerebral ischemia. Neurology. 2017;89:1545–52. 10.1212/WNL.0000000000004487 28904084

